# Population structure and genetic diversity of the perennial medicinal shrub *Plumbago*

**DOI:** 10.1093/aobpla/plv048

**Published:** 2015-05-08

**Authors:** Sayantan Panda, Dhiraj Naik, Avinash Kamble

**Affiliations:** 1 Department of Botany, Savitribai Phule Pune University, Ganeshkhind, Pune 411007, India; 2 Department of Environmental Sciences, Indian Institute of Advanced Research, Koba Institutional Area, Gandhinagar 382007, India

**Keywords:** Genetic diversity, molecular markers, *Plumbago zeylanica*, population structure

## Abstract

Knowledge of the natural genetic variation and structure in a species is important for developing appropriate conservation strategies. As genetic diversity analysis among and within populations of *Plumbago* *zeylanica* remains unknown, we aimed (i) to examine the patterns and levels of morphological and genetic variability within/among populations and ascertain whether these variations are dependent on geographical conditions; and (ii) to evaluate genetic differentiation and population structure within the species. A total of 130 individuals from 13 populations of *P. zeylanica* were collected, covering the entire distribution area of species across India. The genetic structure and variation within and among populations were evaluated using inter-simple sequence repeat (ISSR) and randomly amplified DNA polymorphism (RAPD) markers. High levels of genetic diversity and significantly high genetic differentiation were revealed by both the markers among all studied populations. High values of among-population genetic diversity were found, which accounted for 60 % of the total genetic variance. The estimators of genetic diversity were higher in northern and eastern populations than in southern and western populations indicating the possible loss of genetic diversity during the spread of this species to Southern India. Bayesian analysis, unweighted pair group method with arithmetic average cluster analysis and principal coordinates analysis all showed similar results. A significant isolation-by-distance pattern was revealed in *P. zeylanica* by ISSR (*r* = 0.413, *P* = 0.05) and RAPD (*r* = 0.279, *P* = 0.05) analysis. The results obtained suggest an urgent need for conservation of existing natural populations along with extensive domestication of this species for commercial purpose.

## Abstract (precis)

Knowledge of the natural genetic variation and structure in a species is important for developing appropriate conservation strategies. We aimed to examine (i) the patterns and levels of morphological and genetic variability within/among populations of *Plumbago zeylanica* and ascertain whether these variations are dependent on geographical conditions; and (ii) evaluated genetic differentiation and population structure within the species. ISSR and RAPD markers were used to study the genetic structure and variation within and among populations collected from 13 widespread regions in India. High levels of genetic diversity and significantly high genetic differentiation were revealed by both the markers among all studied populations.

## Introduction

An understanding of the patterns of genetic variation within and among populations of medicinal plants is essential for devising optimum genetic resource management strategies for their conservation, sustainable utilization and genetic improvements. Natural populations of medicinal plant species are extensively exploited due to their heavy demands. In such cases, long-term survival as well as semi-domesticated nature of many medicinal plants depends on the maintenance of sufficient genetic variability within and among populations to accommodate new selection pressures exerted by continuous environmental changes (Barrett and Kohn 1991). Genetic diversity maintained in a plant species would be influenced by many processes, such as the long-term evolutionary history and the characteristics of the species, including genetic drift, gene flow, and reproductive mode and mating system (Hamrick and Godt 1996). Thus, an accurate estimate of genetic diversity of medicinal plant species is influenced by many processes such as the long-term evolutionary process as well as information useful for developing conservation plans to preserve genetic diversity (Falk and Holsinger 1991).

Medicinal plants in India are gaining much attention and are being cultivated widely by the farmers but many of them are still in semi-domesticated nature. Several studies have examined the effects of cultivation on the genetic diversity of crop plant species and forest tree species populations in India (Bahulikar *et al*. 2004; Shaanker *et al*. 2004; Bodare *et al*. 2013; Harish *et al*. 2014). Unfortunately, a handful of studies have examined genetic variation of *Plumbago* and other Plumbaginaceae family members in India inspite of their high economical benefit (Britto *et al*. 2009; Ding *et al*. 2012; Haji *et al*. 2014), and none of them have examined wide-scale genetic structure of *P. zeylanica*. Moreover, very little is known about the impact of environmental conditions such as latitude, longitude and other meteorological variables on genetic structure of this species (Britto *et al*. 2009; Haji *et al*. 2014).

*Plumbago zeylanica*, a perennial shrub of family Plumbaginaceae, is widely dispersed in wild throughout India and has also been introduced as a plantation species. It is native to warmer tropical and sub-tropical regions of world, grows naturally in India, Sri Lanka and in South West Asia (Pant *et al*. 2012). In the recent decades, Plumbago is widely spread in tropical and sub-tropical regions of Australia, Asia and Africa (Tilak *et al*. 2004; Jamal *et al*. 2014) including Ethiopia (Tilak *et al*. 2004). It occurs in deciduous woodland, savannas and scrub forest with an elevation of 300–2000 m. Plant consists of slender stems with thin, glabrous and ovate leaves. The flowers of *P. zeylanica* are dioecious and the pollination is primarily carried out by insect and wind. They are characterized by having a tube-shaped calyx with glandular trichomes secreting sticky mucilage. This plant exhibits both sexual reproduction and clonal growth by rooted shoots (Pant *et al*. 2012). As a traditional Indian medicinal shrub, *P. zeylanica* has a variety of important biological functions, such as inhibiting tumour cell growth, anti-ulceration, anti-deyspepsic and enhancing immunity (Tilak *et al*. 2004; Jamal *et al*. 2014), and has been extensively used to treat chronic diseases. The propagation of *P. zeylanica* seems to be unpredictable due to poor seed viability, improper seed germination and lower seedling recruitment in field conditions are also reported (Pant *et al*. 2012). With the growth of commercial demand in recent years, excessive exploitation has shrunk the natural resource of this species to a narrow distribution, and its survival has been seriously threatened. Previous studies have mainly focused on the resource distribution, its morphological characteristics, dynamics and pharmacological properties (Tilak *et al*. 2004; Hafeez *et al*. 2012, 2013; Jamal *et al*. 2014). Therefore, to formulate conservation strategies for existing natural populations, we aimed to assess the genetic diversity and differentiation between and among populations of *Plumbago* by using randomly amplified polymorphic DNA (RAPD) and inter-simple sequence repeats (ISSR) markers which are widely used because of low cost, easy access and high polymorphic nature. Unlike SSRs these markers do not require prior knowledge of genome sequence. Despite limitations regarding reproducibility of RAPD, combination of ISSR and RAPD markers has been used for understanding population genetic diversity and structure in a number of species (Naik *et al.* 2009; Ding *et al*. 2013; Harish *et al*. 2014).

We focused on large-scale population genetic analysis of *P. zeylanica* using RAPD and ISSR markers to (i) evaluate the wide-range genetic structure of 13 populations selected to cover its distribution across India, (ii) infer relationship between latitude and the components of genetic variation in *P. zeylanica* populations, (iii) compare the population genetic structure in *P. zeylanica* populations using two dominant markers and (iv) provide necessary information for developing conservation strategies for this endangered medicinal shrub.

## Methods

### Ethics statement

No national permissions were required for this study as it did not involve critically endangered or protected species. No specific permissions were required to access the study sites; the collections were made on public lands.

### Study sites and plant sample collection

Healthy seedlings of *P. zeylanica* were randomly selected from each population site covering an area of 50 km and collected during the month of October–December 2010. Data on the coordinates and altitudes of all the population sites are presented **[see Supporting Information—Table S1]**. All the plants collected from different regions were established in the poly house. Authentication of all the plant specimens was done at Botanical Survey of India (BSI), Pune, Maharashtra, India. A total of 13 natural populations of *P. zeylanica* were sampled across four different regions of India, which represented a wide geographic distribution in a range from 97 to 801 m in elevation and 30–394 cm in annual rainfall **[see Supporting Information—Table S1 and Fig. S1]**. To examine the latitudinal pattern of genetic variation within the species, populations were grouped into northern, southern, western and eastern sectors. Ten quantitative and three qualitative morphological traits were measured and examined in 130 individuals that were genotyped **[see Supporting Information—Table S2]**. Ten quantitative morphological descriptors were selected from the International Plant Genetic Resources Institute (IPGRI) descriptors (IPGRI 1993) in the studied populations.

### Genomic DNA extraction

Genomic DNA extraction was carried out using CTAB procedure (de la Cruz *et al*. 1997) with minor modifications. DNA concentration was determined by comparing the intensity of the ethidium bromide stained bands with that of similarly stained bands of known amount of Lambda DNA (Fermentas, USA). The concentration of each DNA sample was made to 10 ng μL^−1^.

### RAPD and ISSR genotyping

Thirty-three ISSR primers from primer set no. 9 (University of British Columbia, Canada) and 50 RAPD primers (Operon, Eurofins Genomics, India) were selected for this study based on the presence of clear, repeatable and polymorphic amplified bands. The amplification was carried out in 20 μL reaction volume and consisted of 0.1 mM of each dNTP, 1 U *Taq* polymerase, 1× of *Taq* polymerase buffer, 1.6 mM MgCl_2_ (Fermentas, USA) and 20 ng genomic DNA. DNA amplification was performed in a thermocycler (Corbett Research, Australia) programmed for an initial denaturation at 94 °C for 5 min, 44 cycles of denaturation at 94 °C for 1 min, annealing at 50 °C/37 °C (for ISSR and RAPD primers, respectively) for 45 s and extension at 72 °C for 1 min and a final extension at 72 °C for 10 min. The amplified products were separated on 2 % agarose gel and stained with ethidium bromide (2 μg mL^−1^). The reproducibility of DNA amplification profiles was tested by repeating the polymerase chain reactions (PCRs) twice with 20 of the 33 selected ISSR primers and 20 of the 50 selected RAPD primers.

### Data analysis

#### Morphological structure of *Plumbago* populations

To describe the structure of individual morphological diversity, a dissimilarity matrix was computed based on the 13 morphological traits. Data for each quantitative trait was scored from 10 randomly chosen plants. From these measurements, the mean, standard deviation (SD) and coefficient of variation (CV) were calculated for each morphological character. The morphological similarity among individuals was then assessed by a principal coordinates analysis (PCoA) using the R package *ade4*.

#### Allelic loci scoring

Reproducible and well-defined bands obtained after PCR amplification using each RAPD and ISSR primers were scored as 1 or 0 for the presence or absence of bands and a binary matrix was generated for RAPD and ISSR markers. Based on the binary data matrix, we estimated the total number of polymorphic loci and percentage polymorphic loci per primer combination.

#### Genetic diversity analysis using RAPD and ISSR markers

The genetic diversity parameters were calculated for each population and for each marker using POPGENE version 1.32 (Yeh *et al*. 2000). The percentage of polymorphic bands (PPB), Nei's gene diversity (*H*) (Nei 1973), Shannon's index, Nei's unbiased genetic distance, Nei's genetic differentiation index among populations (*G*_ST_) and gene flow (*N*_m_) was estimated using POPGENE. The obtained genetic distance matrix was then used to construct the dendrogram using the unweighted pair group method with arithmetic average (UPGMA) algorithm MEGA version 6.0.5 (Tamura *et al*. 2013). To assess percent distribution of genetic variation among and within populations, a hierarchical analysis of molecular variance (AMOVA) was performed using GenAlEx 6.2 software (Excoffier *et al*. 1992; Peakall and Smouse 2012). Genetic distance was tested against geographic distance by Mantel test with 999 random permutations using GenAlEx 6.2 software (Excoffier *et al*. 1992). The effect of latitude on genetic diversity was analysed by He, PPB for each population for both the markers. In addition, AMOVA was conducted to estimate genetic variation among latitudinal sectors and a linear regression was tested against latitude using SigmaPlot, version 10.0, considering He and PPB as dependent variables.

#### Population structure analysis

The Bayesian clustering method was implemented to deduce population structure using STRUCTURE 2.2.3 software (Pritchard *et al*. 2000; Falush *et al*. 2003). STRUCTURE performs Bayesian assignments of individuals to a given number of genetically homogenous clusters (*K*) of populations. Twenty replications of each proposed *K* value (from *K* = 1 through *K* = 20) were investigated under no-admixture ancestry and the correlated allele frequencies model by running 100 000 iterations of each *K*, with a burn-in length of 100 000 iterations. To assist the determination of optimal *K*, Δ*K* was estimated as described (Evanno *et al*. 2005). The probability distribution [ln *P*(*D*)] and Δ*K* were retrieved from the STRUCTURE HARVESTOR software (Earl and vonHoldt 2012). Bar chart for the proportion of the member coefficient of each individual for each *K* was summarized using CLUMPP (Jakobsson and Rosenberg 2007) and visualized in DISTRUCT (Rosenberg 2004).

## Results

### Morphological variability

In multidimensional analysis of morphological data matrix containing quantitative and qualitative characters, sampled population of *P. zeylanica* was significantly distinguishable and was quite variable representing high levels of inter- and intra-population variation **[see Supporting Information—Fig. S2]**. The principal component analysis (PCA) represented that the first two components, which had eigenvalue higher than 1, denotes the total of 77.6 % of whole phenotypic variability, contributing to all the variables to the morphological diversity of sampled populations **[see Supporting Information—Fig. S3 and Table S2]**. The most discriminative quantitative characters were length of the inflorescence axis, number of inflorescence per vine, number of flower per inflorescence, distance between two adjacent flowers and length of petal based on correlation of these characters with PC1. Principal component analysis indicated that the habit type and trichome colour on the inflorescence axis were the two best qualitative diagnostic characters. Both traits were highly correlated with first PCA axis, and showed a semi-overlapping pattern of variation among the sampled populations.

### ISSR and RAPD polymorphism

A total of 130 individuals belonging to 13 populations of *P. zeylanica* were surveyed. Among and within studied populations generated a total of 229 fragments by using 20 selected ISSR primers, of which 169 (73.8 %) were polymorphic **[see Supporting Information—Table S3]**. Each primer amplified 10–19 bands with an average of 14.6. The size of the amplified fragments ranged from 200 to 2000 bp. In general, ISSR variation within populations was very low and varied erratically across localities (Table 1).

**Table 1.** Genetic diversity within populations of *P. zeylanica* using ISSR markers. PPB, percentage of polymorphic bands.

Populations	Region	Effective number of alleles (*n*_e_)	PPB (%)	Nei's genetic diversity (*h*)	Shannon's information index (*I*)
Solan, Himachal Pradesh	North	1.03	08.30	0.021	0.033
Panipat, Haryana	North	1.04	10.48	0.028	0.045
Ananthagiri, Vikarabad, Andhra Pradesh	South	1.04	11.35	0.028	0.047
Tirupati, Chittor, Andhra Pradesh	South	1.04	10.92	0.026	0.044
Kolli, Salem, Tamilnadu	South	1.02	09.17	0.018	0.032
Coimbatore, Tamilnadu	South	1.06	15.72	0.042	0.069
RFRI Campus, Jorhat, Assam	East	1.01	06.55	0.012	0.022
NBU Campus, Siliguri, West Bengal	East	1.02	06.99	0.016	0.027
Kadma, Bankura, West Bengal	East	1.03	11.79	0.024	0.042
Ajra-Amboli, Kolhapur, Maharashtra	East	1.03	09.61	0.022	0.037
JNVU Campus, Jodhpur, Rajasthan	West	1.05	13.10	0.035	0.057
Ellora, Aurangabad, Maharashtra	West	1.05	13.54	0.036	0.058
Shendi, Bhandardara, Maharashtra	West	1.06	15.28	0.038	0.063
Average		1.04	11.0	0.03	0.04
Species-level		1.44 (0.347)	73.8	0.26 (0.173)	0.41 (0.230)

The RAPD analysis yielded a total of 232 loci for 130 individuals generated by using 20 selected primers, of which 78.9 % (183 fragments) were polymorphic between individuals **[see Supporting Information—Table S4]**. In comparison to ISSR profiling, RAPD variation within populations was higher in *P. zeylanica* and varied across the populations (Table 2).

**Table 2.** Genetic diversity within populations of *P. zeylanica* using RAPD markers. PPB, percentage of polymorphic bands.

Populations	Region	Effective number of alleles (*n*_e_)	PPB (%)	Nei's genetic diversity (*h*)	Shannon's information index (*I*)
Solan, Himachal Pradesh	North	1.07	09.05	0.040	0.057
Panipat, Haryana	North	1.09	11.21	0.049	0.070
Ananthagiri, Vikarabad, Andhra Pradesh	South	1.09	11.64	0.051	0.073
Tirupati, Chittor, Andhra Pradesh	South	1.08	10.34	0.045	0.065
Kolli, Salem, Tamilnadu	South	1.06	08.62	0.037	0.054
Coimbatore, Tamilnadu	South	1.11	14.22	0.062	0.089
RFRI Campus, Jorhat, Assam	East	1.05	06.47	0.028	0.040
NBU Campus, Siliguri, West Bengal	East	1.06	07.76	0.034	0.048
Kadma, Bankura, West Bengal	East	1.09	12.50	0.054	0.078
Ajra-Amboli, Kolhapur, Maharashtra	East	1.07	09.05	0.039	0.056
JNVU Campus, Jodhpur, Rajasthan	West	1.11	13.79	0.060	0.087
Ellora, Aurangabad, Maharashtra	West	1.09	12.50	0.054	0.078
Shendi, Bhandardara, Maharashtra	West	1.12	15.95	0.069	0.100
Average		1.08	11.0	0.05	0.07
Species-level		1.52 (0.329)	78.9	0.31 (0.155)	0.47 (0.197)

### Genetic diversity

Based on ISSR profiling, genetic diversity of the species across all the populations was with an average *H* = 0.034 (SE = 0.001) and Ne, PPB and I were on average 1.04 (ranged from 1.01 to 1.06), 11 (ranged from 6.55 to 15.28) and 0.04 (ranged from 0.02 to 0.06), respectively (Table 1). As a general pattern, genetic diversity and percent polymorphic bands of *P. zeylanica* populations decreased with increasing latitude **[see Supporting Information—Fig. S4]**. Overall genetic diversity indices showed that the city Coimbatore from southern region of India has the highest diversity and Jorhat from eastern region of India has the lowest. Genetic diversity of western region populations was double compared with eastern region populations (Table 1). Southern and northern region populations showed intermediate genetic diversity when compared with western region populations (Table 1).

Similar to ISSR analysis, RAPD analysis showed decreased genetic diversity and PPBs of *P. zeylanica* populations with increasing latitude **[see Supporting Information—Fig. S4]**. Assuming a Hardy–Weinberg equilibrium, *H* was with an average of 0.051 ± 0.002 whereas Ne, PPB and I were on average 1.08 (ranged from 1.05 to 1.12), 11 (ranged from 6.47 to 15.95) and 0.07 (ranged from 0.04 to 0.10), respectively (Table 2). All genetic diversity indices showed that Jodhpur from western region has the highest diversity and Jorhat towards the east has the lowest. Genetic diversity of western region populations was double compared with eastern region populations (Table 2). Southern and northern region populations showed intermediate genetic diversity when compared with western region populations (Table 2).

### Genetic differentiation and gene flow

Distribution of total genetic variation by nested AMOVA for ISSR dataset revealed that most of the total variance is attributable to genetic variation among populations (61 %) (Table 3). However, 37 and 2 % of the variance was partitioned within populations and among regions, respectively. Analysis of molecular variance revealed a low level of genetic differentiation, but this was highly significant (*P* < 0.001) between within and among populations (Table 3). Pronounced level of genetic differentiation among populations (*ϕ*_ST_ = 0.627, *P* < 0.001) and limited estimated gene flow between populations (*N*_m_ = 0.06) was observed. In RAPD dataset for the same populations, nested AMOVA showed that vast majority of variance (57 %) was among populations, while 43 and 0 % of the variance was partitioned within populations and among regions, respectively (Table 4). A significant level of genetic differentiations was observed within (*ϕ*_ST_ = 0.572, *P* < 0.001) and among (*ϕ*_ST_ = 0.571, *P* < 0.001) populations, while low among-population gene flow (*N*_m_ = 0.09) was observed, using RAPD dataset.

**Table 3.** Analysis of molecular variance for 130 individuals of *P. zeylanica* using ISSR markers, significance tests after 1000 random permutations. df, degrees of freedom; SSD, sum of squares; TVP, total variance component.

Source of variation	df	SSD	Variance components	TVP (%)	*P*-value
Variance among region	3	790.71	0.75	2	0.007
Variance among populations (variance within region)	9	2157.27	22.62	61	0.001
Variance within populations	117	1574.50	13.45	37	0.001
Total	129	4522.48	36.83	100

**Table 4.** Analysis of molecular variance for 130 individuals of *P. zeylanica* using RAPD markers, significance tests after 1000 random permutations. df, degrees of freedom; SSD, sum of squares; TVP, total variance component.

Source of variation	df	SSD	Variance components	TVP (%)	*P*-value
Variance among region	3	706.36	0.00	0	0.656
Variance among populations (variance within region)	9	2154.14	22.27	57	0.001
Variance within populations	117	1946.20	16.63	43	0.001
Total	129	4806.70	38.90	100	

### Genetic relationship and structuring

POPGENE analysis revealed that Nei's unbiased genetic distance ranged from 0.151 (Coimbatore vs. Aurangabad) to 0.633 (Aurangabad vs. Jorhat) in ISSR dataset, while with RAPD dataset, Nei's unbiased genetic distance ranged from 0.584 (Kadma vs. Jorhat) to 0.913 (Ananthagiri to Aurangabad). The UPGMA tree based on Nei's unbiased genetic distance resolved them into three clusters **[see Supporting Information—Figs S5 and S6]** mainly in correspondence with eastern and western region. The Siliguri population formed a sole group, and the remaining populations formed the other group, which can be subdivided into two subgroups. Ananthagiri and Aurangabad populations, which showed highest similarity, were grouped together. This cluster further grouped with Coimbatore and Ajra. These four populations, which were from Deccan Platue, formed one major cluster. Populations close to Himalaya formed a separate cluster, which showed bootstrap values of 56 %. Bootstrap values ranged from 44 to 95 % for each population cluster. These values indicated that each population showed high confidence limits for clustering. Bootstrap values at the nodes joining two different populations ranged from 53 to 89 %. While Ananthagiri and Aurangabad populations showed the bootstrap value of 95 %, hierarchical clustering of these with Coimbatore and Ajra populations showed bootstrap values of 86 and 84 %, respectively. Similarly, the bootstrap value for the cluster of Solan and Panipat populations was 56 %. Clustering of these with Siliguri showed moderately low bootstrap values of 44 %.

The two-dimensional PCoA for 230 individuals in 13 populations based on ISSR dataset accounted for 28.15 % (axis 1) and 18.43 % (axis 2) of total variance, respectively **[see Supporting Information—Fig. S7]**. As expected, Silguri, Jorhat, Solan, Kadma, Panipat and Bhandarada populations occupied similar position along the axis 1. The other populations such as Ajra, Aurangabad, Coimbatore and Ananthagiri have similar genetic similarity.

**Figure 1.**
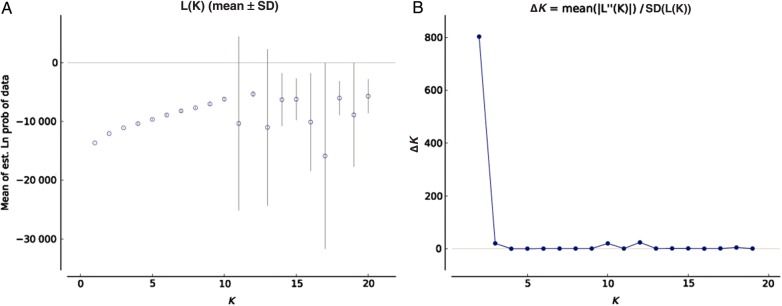
Line graphs from the STRUCTURE model of ln *P*(*D*) (a measure of the natural logarithm of the posterior probability, *P* of the data, *D*) and Δ*K* for sampled *P. zeylanica* populations using ISSR marker (*P*), where *K* is the hypothesized number of populations. (A) The mean values of ln *P*(*D*) and SD from 10 runs for each value of *K* = 1–20. (B) The distributions of Δ*K* over *K* = 1–20.

**Figure 2.**
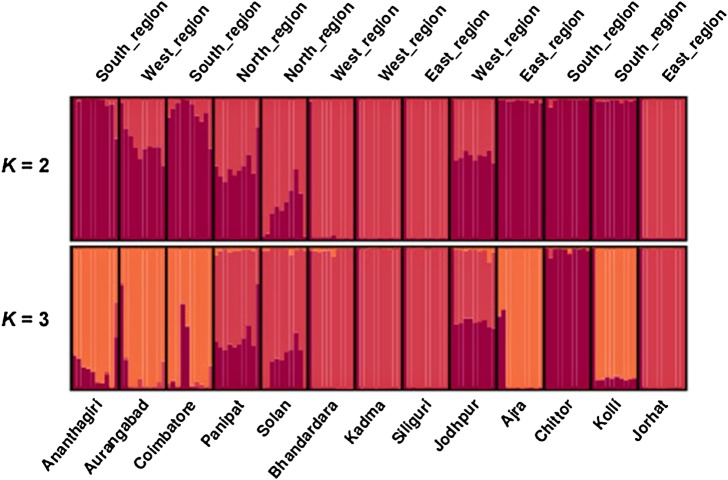
Genetic structure of *P. zeylanica* populations inferred from Bayesian clustering method (Pritchard *et al*. 2000) STRUCTURE plot of 130 wild *P. zeylanica* individuals using ISSR markers. The *y*-axis shows the proportion membership into the various clusters. Each coloured vertical bar represents a single individual and the 10 individuals from each of the 13 sampled populations are grouped together. Vertical black bars have been included as visual separators between the populations.

In the STRUCTURE analysis using ISSR dataset, the real *K* value with the highest value of Δ*K* for the 130 individuals was 2 (Fig. 1) followed by levelling off and accompanied by an increase in variance. A diagnostic for true value of *K* is a decrease in slope and increase in variance of ln *P*(*D*) (Fig. 1). The proportion of each individual in each population assigned into two sub-clusters (Clusters I and II) (Fig. 2), resulted in agreement with UPGMA dendrogram **[see Supporting Information—Fig. S5]**. Several single populations were assigned to specific clusters for higher values of *K* (Fig. 2). Although several nodes were poorly supported by bootstraps, the results of the UPGMA tree showed a similar pattern as that of the STRUCTURE analysis **[see Supporting Information—Fig. S5]** (Fig. 2).

**Figure 3.**
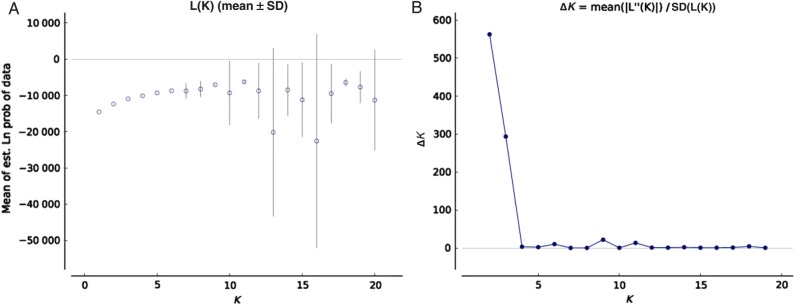
Line graphs from the STRUCTURE model of ln *P*(*D*) (a measure of the natural logarithm of the posterior probability, *P* of the data, *D*) and Δ*K* for sampled *P. zeylanica* populations using RAPD marker (*P*), where *K* is the hypothesized number of populations. (A) The mean values of ln *P*(*D*) and SD from 10 runs for each value of *K* = 1–20. (B) The distributions of Δ*K* over *K* = 1–20.

**Figure 4.**
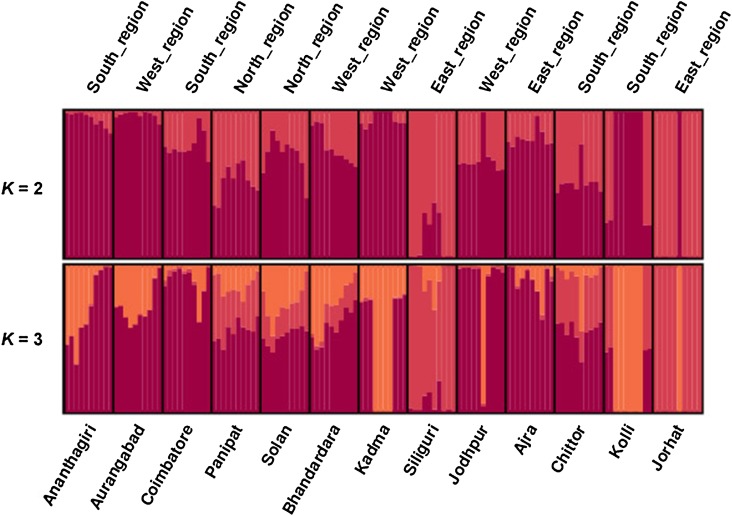
Genetic structure of *P. zeylanica* populations inferred from Bayesian clustering method (Pritchard *et al*. 2000) STRUCTURE plot of 130 wild *P. zeylanica* individuals using RAPD markers. The *y*-axis shows the proportion membership into the various clusters. Each coloured vertical bar represents a single individual and the 10 individuals from each of the 13 sampled populations are grouped together. Vertical black bars have been included as visual separators between the populations.

The population structure of *P. zeylanica* using RAPD dataset inferred using the method of Pritchard *et al*. (2000) showed a spatial pattern of genetic distances among the populations which was similar to the results of UPGMA dendrogram. On the basis of the method of Evanno *et al*. (2005), all the analysed genotypes were split into *K* = 2 groups (Fig. 3). Several single populations were assigned to specific clusters for higher values of *K* (Fig. 3). However, the clustering patterns for values *K* > 4 showed complicated multimodality, which could be because of assignment of individuals to clusters in inconsistent between runs. This indicates some of the models were difficult to fit into the data. However, some populations were not fully supported with any of the two clusters; rather, it appears to be admixed **[see Supporting Information—Fig. S4]**. Although several nodes were poorly supported by bootstraps, the results of the UPGMA tree showed a similar pattern as that of the STRUCTURE analysis **[see Supporting Information—Fig. S6]** (Fig. 4). The Mantel's test result showed a positive correlation between geographic and genetic distance with significance detected using ISSRs (*r* = 0.413, *P* = 0.05, 999 permutations), and by RAPDs (*r* = 0.279, *P* = 0.05, 999 permutations) (data not shown).

## Discussion

This study provides a first report of broad survey of genetic variation in *P. zeylanica* along the latitudinal gradient in India. There is a substantial variation in environmental conditions along the geographic range which encompasses the edaphic conditions supporting natural vegetation of *P. zeylanica* and climatic conditions. Under changing environmental conditions, genetic diversity is crucial for effective management and developing conservation strategies for valuable, endemic and medicinally important species *P. zeylanica*. To understand the extent of genetic diversity, genetic structure and differentiation among *P. zeylanica* populations occurring in different geographic regions of India, two PCR-based molecular markers, namely RAPD and ISSR, were selected based on their applicability in other medicinal plant taxa that were used for both inter- and intra-population analysis (Pither *et al*. 2003; Juchum *et al*. 2007; Naik *et al*. 2009).

Based on morphometric data, it was observed that flower-oriented traits showed distinct variability among the populations of *P. zeylanica* **[see Supporting Information—Table S2 and** **Fig. S8****]**. The UPGMA analysis based on average taxonomic distance among the populations produced two distinct clusters. Examination of character coefficient revealed that many of the original variables were strongly and positively correlated with PC1 including flower size and shape, colour of trichomes on sepal, leaf area, stem height and plant habits. Principal component analysis showed that populations from different geographic locations with higher mean annual precipitation tended to be large in size in all floral and vegetative traits **[see Supporting Information—Fig. S1]**. However, other traits of supposedly taxonomic importance such as flower shape and leaf area exerted only minor influence on PC1 **[see Supporting Information—Fig. S8]**. Two distinctive colours of glandular trichomes were noted, namely semi-transparent and purple within the populations studied, which might have an adaptive response against phytophagous insect herbivory (Jaime *et al*. 2013). Plumbaginaceae is considered to be related to Droceraceae (Jaime *et al*. 2013) and it was reported that glandular trichomes in the genus *Plumbago* is capable of secreting proteases in response to chemical stimulation but not in a way that would be typical of a true carnivore (Jaime *et al*. 2013). Beside the genus *Plumbago*, the genus from same family *Limonium* shows many leaf and gland characteristics common to the Plumbago and the Caryophyllales carnivores that might be expected in the last common ancestor with the carnivores (Meimberg *et al*. 2000). The observed variation in herbivore pressure among taxa likely caused by habitat differentiation might have played a role in trait differentiation through divergent selection or may be due to interaction with some specific insect. The change in colour of trichomes might be to attract insects (Healy *et al*. 2009), as purple colour is more attractive over semi-transparent and this might be in reference to nutrient levels in soil (Pawar *et al*. 2008). In present study, populations with purple trichomes were mainly from Deccan platue region namely Coimbatore, Ananthagiri, Ajra and Aurangabad **[see Supporting Information—Fig. S2]**. These regions are known to have nitrogen poor soil as it is formed from igneous rocks (Pawar *et al*. 2008). To overcome this nitrogen deficiency, the populations spread to Deccan platue might have been evolving towards insectivorous habit and the change in trichome colour from semi-transparent to purple might be an adaptive trait in attracting more insects.

To correlate phenotypic variation with molecular phylogeny, ISSR and RAPD markers were used because they can detect very low levels of genetic variation, making them powerful genetic markers which have been used in genetic diversity and population genetic studies of wild plants (Pither *et al*. 2003; Bahulikar *et al*. 2004; Juchum *et al*. 2007; Naik *et al*. 2009; Bodare *et al*. 2013). In the present study, we have shown that these markers revealed a significant genetic variation among geographically separated sub-populations of *P. zeylanica* in Indian regions. Inter-simple sequence repeats and RAPDs also revealed diversity within each population. The obtained results based on heterozygosity data for both the markers in accordance with various studies in other wild plant species suggest the line of evidence that inter-population genetic diversity is higher than the intra-populations, suggesting that a significant genetic variation is maintained due to outcrossing events in this species.

In 13 populations of *P. zeylanica* studied, we found that Shannon's index of genetic diversity was estimated to be an average 0.04 (by ISSRs) and 0.07 (by RAPDs) (Tables 1 and 2). These values can be compared with other plant species with similar life histories. Our survey of 13 populations of *P. zeylanica* revealed high genetic diversity [ISSR and RAPD analysis revealed band polymorphism = 73.8 and 78.9 %, Nei's gene diversity (*H*) = 0.26 and 0.31, Shannon's index (*I*) = 0.41 and 0.47, respectively] at the species level (Tables 1 and 2). Similar results were observed in genetic diversity studies of southern India populations of *P. zeylanica* (Britto *et al*. 2009; Haji *et al*. 2014). Limited sample size was the major limitation of the previous study; moreover, Haji *et al.* (2014) did not consider the geographical distribution of the species. Genetic diversity at the population level was observed to be comparatively lower [from ISSR and RAPD assay band polymorphism = 11 % for both, Nei's gene diversity (*H*) = 0.03 and 0.05, Shannon's index (*I*) = 0.04 and 0.07).

High level of among-population genetic differentiation was revealed in *P. zeylanica* population using both ISSR and RAPD markers (*G*_ST_ = 0.90 and 0.84 from ISSR and RAPD analysis, respectively), indicating that the populations were subjected to genetic isolation. Similar results were also reported in many other medicinal and endemic species such as *Torreya jackii* (Li and Jin 2007), *Rhodiola alsia* (Xia *et al*. 2005), and *Rheum palmatuma* and *R. tanguticum* (Wang *et al*. 2012). The higher genetic differentiation of population within a species is driven by various factors such as genetic isolation or genetic drift, pollination and breeding system and geographic distribution range (Young *et al*. 1996; Hogbin and Peakall 1999). The geographic distribution and topographical barriers can lead to difficulties in seed dispersal resulting in limited gene flow among populations (Hamrick and Godt 1996). Mantel test showed a significant isolation-by-distance pattern in *P. zeylanica*, indicating genetic isolation has a significant effect on genetic variation and structure in this species. We also observed that the gene flow between the populations was low (*N*_m_ = 0.06 and 0.09). The sampling sites chosen in this work were geographically widely separated (Solan and Coimbatore = ∼2800 km; Jodhpur and Jorhat = ∼2600 km) and this may account for the high genetic diversity and gene differentiation observed in this species. However, some of the sampling sites (Kolli and Coimbatore) were separated by ∼150 km, and the probable causes underlying genetic diversity of these populations could not be attributed to geographical separation alone. From the present study it is revealed that the populations of *P. zeylanica* from different eco-geographical regions of India were grouped into two major clusters, one with purple trichomes and other with semi-transparent trichomes on sepal, this is also supported by both ISSR and RAPD data (Figs 2 and 4).

Habitat fragmentation is an important cause of alteration in the population structure of plants (Young *et al*. 1996). In our study, the fragmented populations of *P. zeylanica* represented by Jorhat, Siliguri and Kolli populations showed lower diversity (*H* = 0.12, 0.16 and 0.18, respectively, from ISSR analysis and 0.028, 0.034 and 0.037, respectively, from RAPD analysis) when compared with the natural forest represented by Bhandardara population (ISSR and RAPD assay revealed *H* = 0.038 and 0.069, respectively). This could be attributed to a reduction in the gene pool and increased gene differentiation in fragmented forests, which arises due to the presence of fewer individuals in the population and induced inbreeding (Young *et al*. 1996). High levels of gene differentiation were also reported in other species existing in the fragmented habitats. Some of these were also endangered medicinally important species like *Sinopodophyllum hexandrum* (*G*_ST_ = 0.62) (Xiao *et al*. 2006), *Podophyllum hexandrum* (*G*_ST_ = 0.75) (Alam *et al*. 2009), *Saruma henryi* (*G*_ST_ = 0.69) (Zhou *et al*. 2010), *Magnolia officinalis* (*G*_ST_ = 0.67) (Yu *et al*. 2011), indicating that fragmentation, besides bringing about a high level of differentiation in populations, also leads to a reduction in their numbers. A critical number of individuals are required to sustain a population, reduction in the number of individuals below this level affects the sustainability of the species and leads to their elimination (Rajora and Mosseler 2001). Plant species that are not yet endangered, but whose populations have been fragmented due to anthropogenic activities, also show high levels of differentiation such as *Dactylorhiza hatagirea* (*G*_ST_ = 0.25) (Warghat *et al*. 2012) or *Curcuma zedoaria* (Islam *et al*. 2006). *Plumbago zeylanica* belongs to the latter category of plants, as it is largely pollinated by bees and other insects preferentially due to extensive network of sticky glands present on the floral surface. The seed dispersal in *P. zeylanica* populations is due to herbivores, which limits their spread. The limited seed spread and seedling establishment may contribute to a reduced gene flow in this species (Lowe *et al*. 2005). Between- and within-population genetic diversity of the species also depends on the type of pollination and subsequent breeding system. Dioecious species like *Eurya nitida* (HS = 0.13) (Bahulikar *et al*. 2004), as well as other outcrossing species like *Taxus fauna* (HS = 0.12) (Shah *et al*. 2008), and *Changium smyrnioides* (HS = 0.11) (Qiu *et al*. 2004), showed high genetic diversity within populations.

Genetic diversity within a species is shaped over long periods of time through evolutionary genetic processes acting in combination on that species (Rajora and Mosseler 2001). The evolutionary history of *P. zeylanica* is not known, but the species is widely distributed in the different eco-climatic zones of India, which is suggestive of a broad genetic base (Pant *et al*. 2012; Haji *et al*. 2014). Reduction and fragmentation in wild medicinal plants due to over-exploitation in the forest cover could be one of the main causes that led to an increase in genetic differentiation and reduced gene flow between populations.

## Conclusions

The results of the present study suggest that *P. zeylanica* has higher genetic diversity at species and population level as assessed by two different molecular markers however, extraordinarily high among-population genetic differentiation existed in *P. zeylanica*. A larger proportion of genetic variation was observed among populations. A greater effort should be made to preserve all the extant populations and their habitats in the field, especially with those populations with higher genetic diversity. Considering higher demands for raw tissue material and heavy exploitations, these wild *Plumbago* resources have long been subjected to excessive collections. It would be sustainable if plantation of new populations can be established to meet the market demand. This way it can alleviate the excessive collection of natural resources of *P. zeylanica*.

To conclude, the present study generated information useful for developing appropriate conservation strategies which would ensure that there is less anthropogenic destruction of existing habitats, increase in the natural population size, optimization and improvement of cultivation practices ensuring constant supply of plant material without exploiting the natural populations.

## Sources of Funding

Our work was funded by University Grant Commission, New Delhi, India under DRS-SAP III programme.

## Contributions by the Authors

Conceived and designed the experiments: S.P. and A.K. Performed the experiments: S.P. Analysed the data: S.P., D.N., and A.K. Contributed reagents/materials/analysis tools: S.P., D.N. and A.K. Wrote the paper: D.N., A.K. and S.P.

## Conflict of Interest Statement

None declared.

## Supporting Information

The following additional information is available in the online version of this article –

**Table S1.** Sampling details of *P. zeylanica* populations.

**Table S2.** Descriptive statistics of the 13 traits measured on populations collected.

**Table S3.** ISSR primers used for ISSR analysis.

**Table S4.** RAPD primers used for RAPD analysis.

**Figure S1.** Location of 13 sampled *P. zeylanica* populations in India.

**Figure S2.** Types of glandular trichomes on sepal of *P. zeylanica*.

**Figure S3.** Principal component analysis (PCoA) of growth and morphological variables.

**Figure S4.** Regression of genetic diversity (A: He and B: PPB) on latitude (oS) for ISSR (a and b) and RAPD (c and d) marker data and (C: He and D: PPB) of *P. zeylanica* populations.

**Figure S5.** Unrooted dendrogram from the neighbor-joining analysis of ISSR markers.

**Figure S6.** Unrooted dendrogram from the neighbor-joining analysis of RAPD markers.

**Figure S7.** Principal component analysis (PCoA) using ISSR and RAPD data.

**Figure S8.** Types of growth habit observed in *P. zeylanica* populations.

Additional Information
