# Supplementary material for: Population structure and genetic diversity of the perennial medicinal shrub Plumbago
Source: AoB Plants. 2015 May 8;7:plv048. doi: 10.1093/aobpla/plv048 (PMC4501514; doi:10.1093/aobpla/plv048)
Supplement: Additional Information [file supp_7_plv048_index.html]

Population structure and genetic diversity of the perennial medicinal shrub Plumbago — Additional Information 

# Population structure and genetic diversity of the perennial medicinal shrub *Plumbago*

## Additional Information

Additional Information

- Additional Figures - doc file
- Additional Tables - doc file
