# Supplementary material for: Population structure and genetic diversity of the perennial medicinal shrub Plumbago
Source: AoB Plants. 2015 May 8;7:plv048. doi: 10.1093/aobpla/plv048 (PMC4501514; doi:10.1093/aobpla/plv048)

**Figure S1**

Location of 13 sampled *P. zeylanica* populations in India. This map shows location of populations within four latitudinal sectors. Units indicated in kilometers.

**Figure S2**

Types of glandular trichomes on sepal of *P. zeylanica* (A) Semi-transparent trichomes; (B) Purple trichomes.

**Figure S3**

Principal component analysis (PCoA) of growth and morphological variables observed in thirteen populations of *P. zeylanica*.

**Figure S4**

Regression of genetic diversity (A: He and B: PPB) on latitude (^o^S) for ISSR (a & b) and RAPD (c & d) marker data and (C: He and D: PPB) of *P. zeylanica* populations in all sampled populations. He, mean expected heterozygosity; PPB, percentage of polymorphic loci per population.

**Figure S5**

Unrooted dendrogram from the neighbor-joining analysis of ISSR markers showing relationships between 13 sampled populations of *P. zeylanica*. Bootstrap replicate values greater than 50% are shown above the branch.

**Figure S6**

Unrooted dendrogram from the neighbor-joining analysis of RAPD markers showing relationships between 13 sampled populations of *P. zeylanica*. Bootstrap replicate values greater than 50% are shown above the branch.

**Figure S7**

Principal component analysis (PCoA) using ISSR and RAPD data of 130 individuals in thirteen populations of *P. zeylanica*. (A) ISSR (B) RAPD

**Figure S8**

Types of growth habit observed in *P. zeylanica* populations; two distinct growth types were observed (A) Struggling erect; (B) Clear erect.


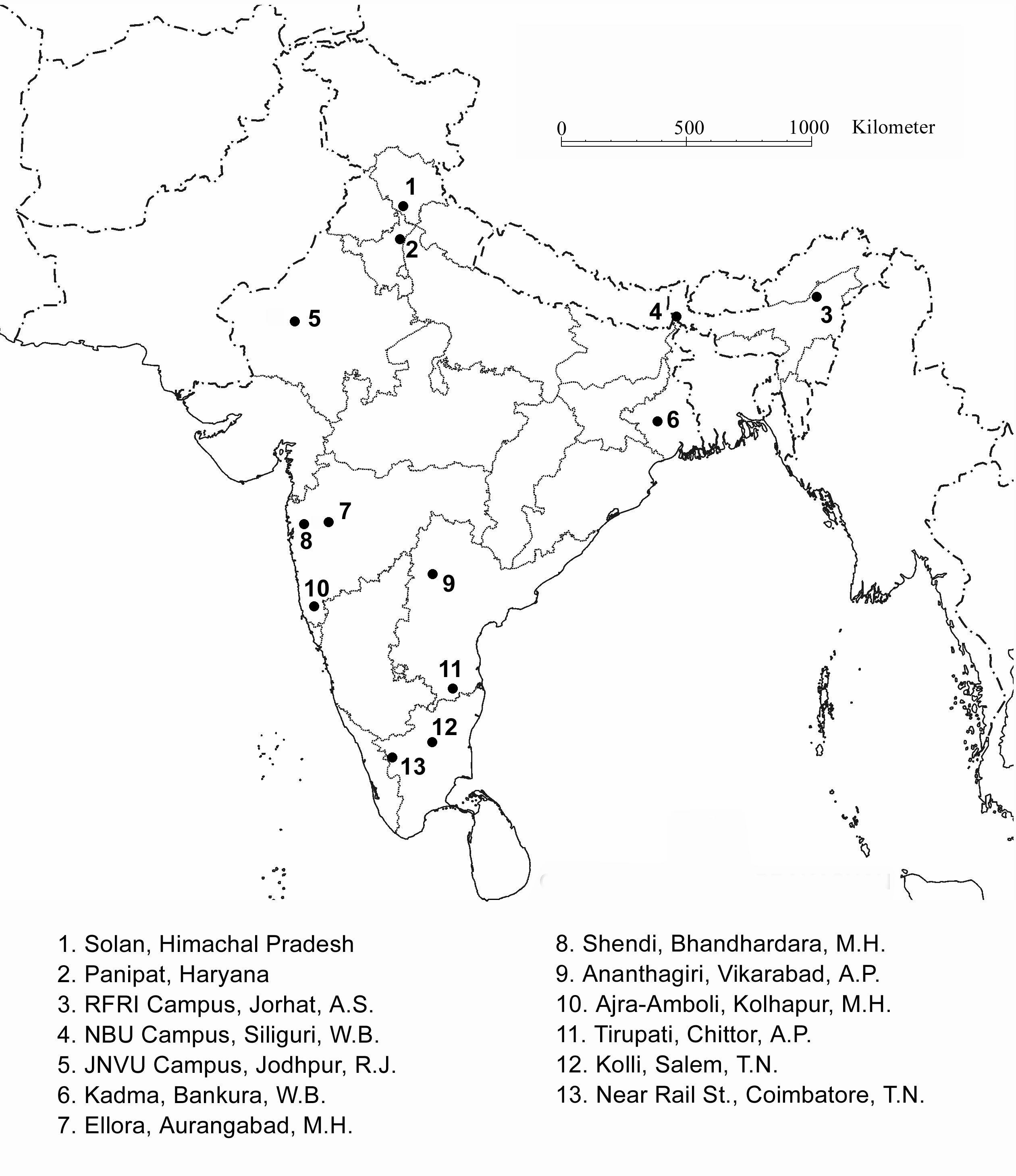


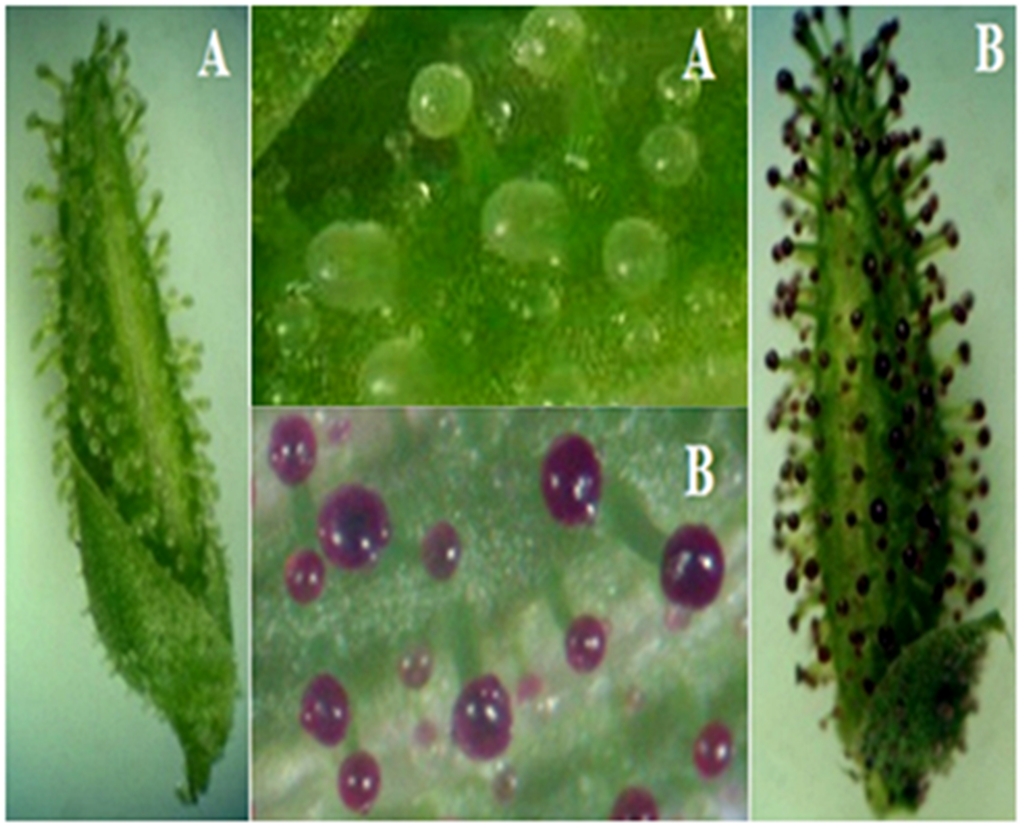


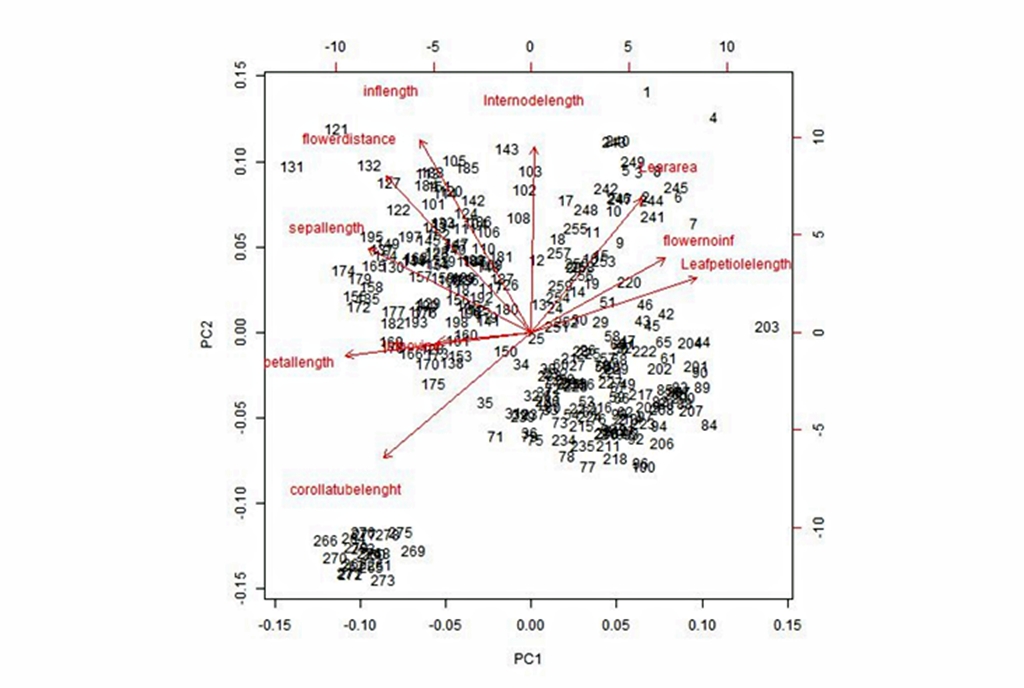


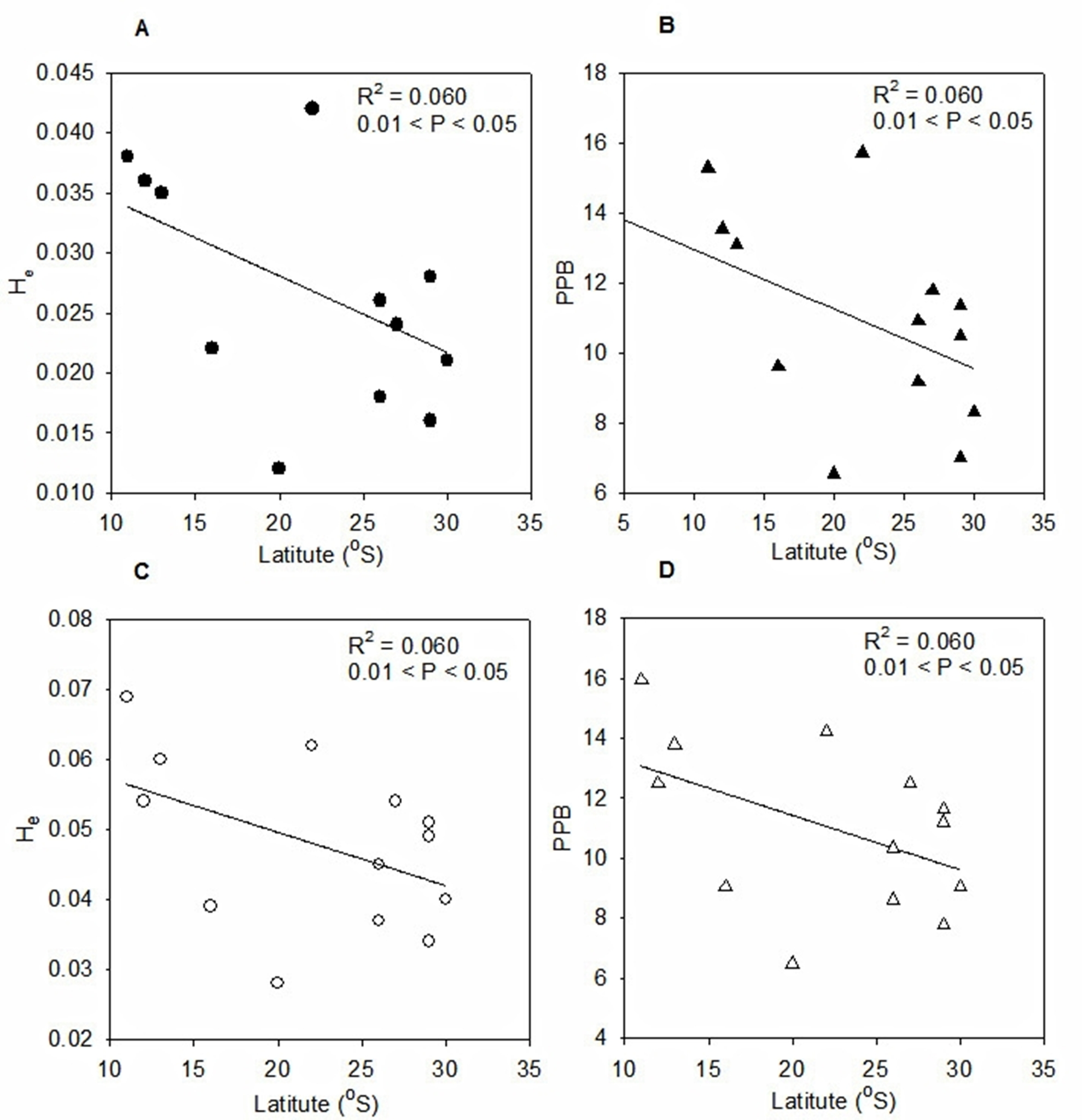


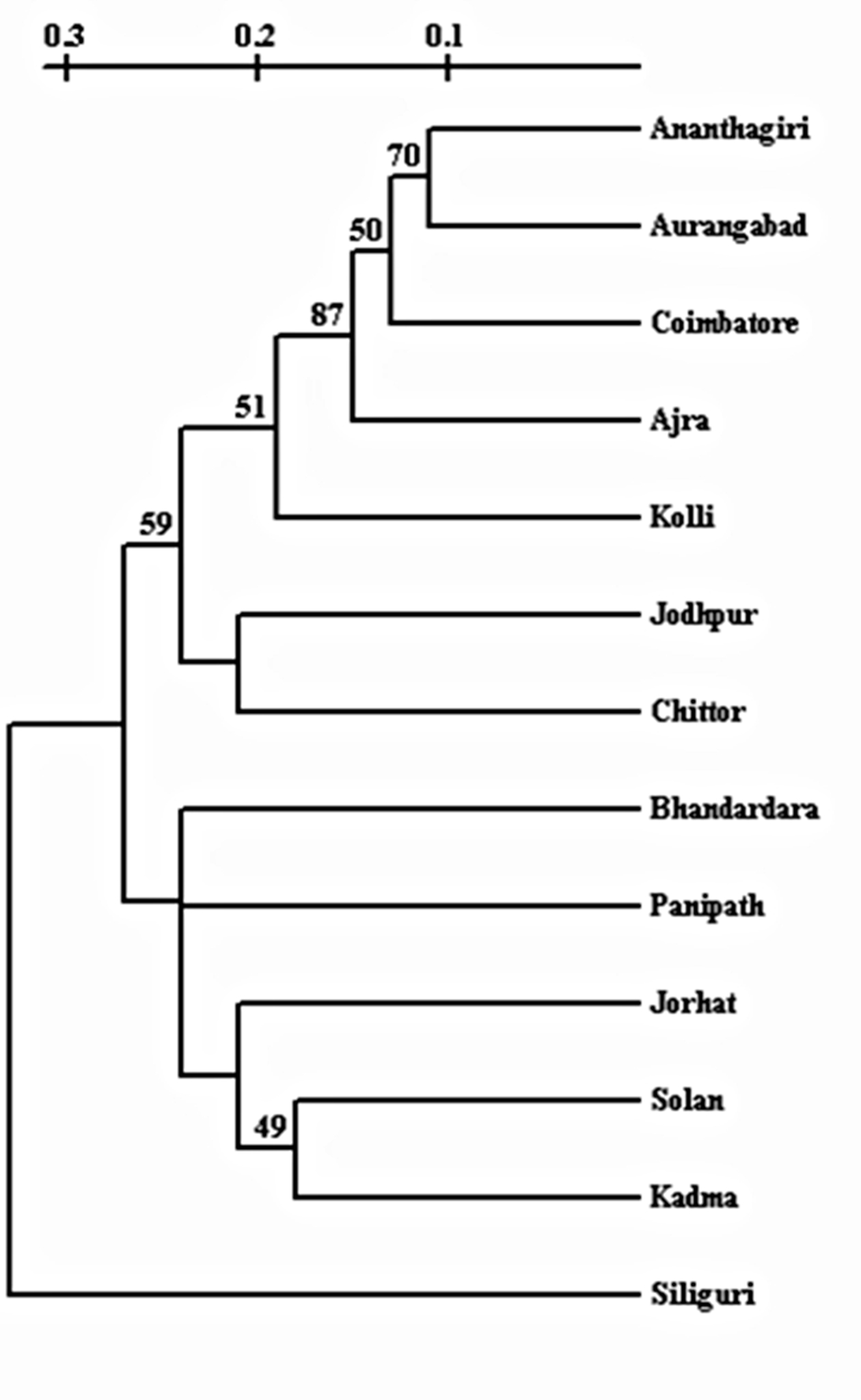


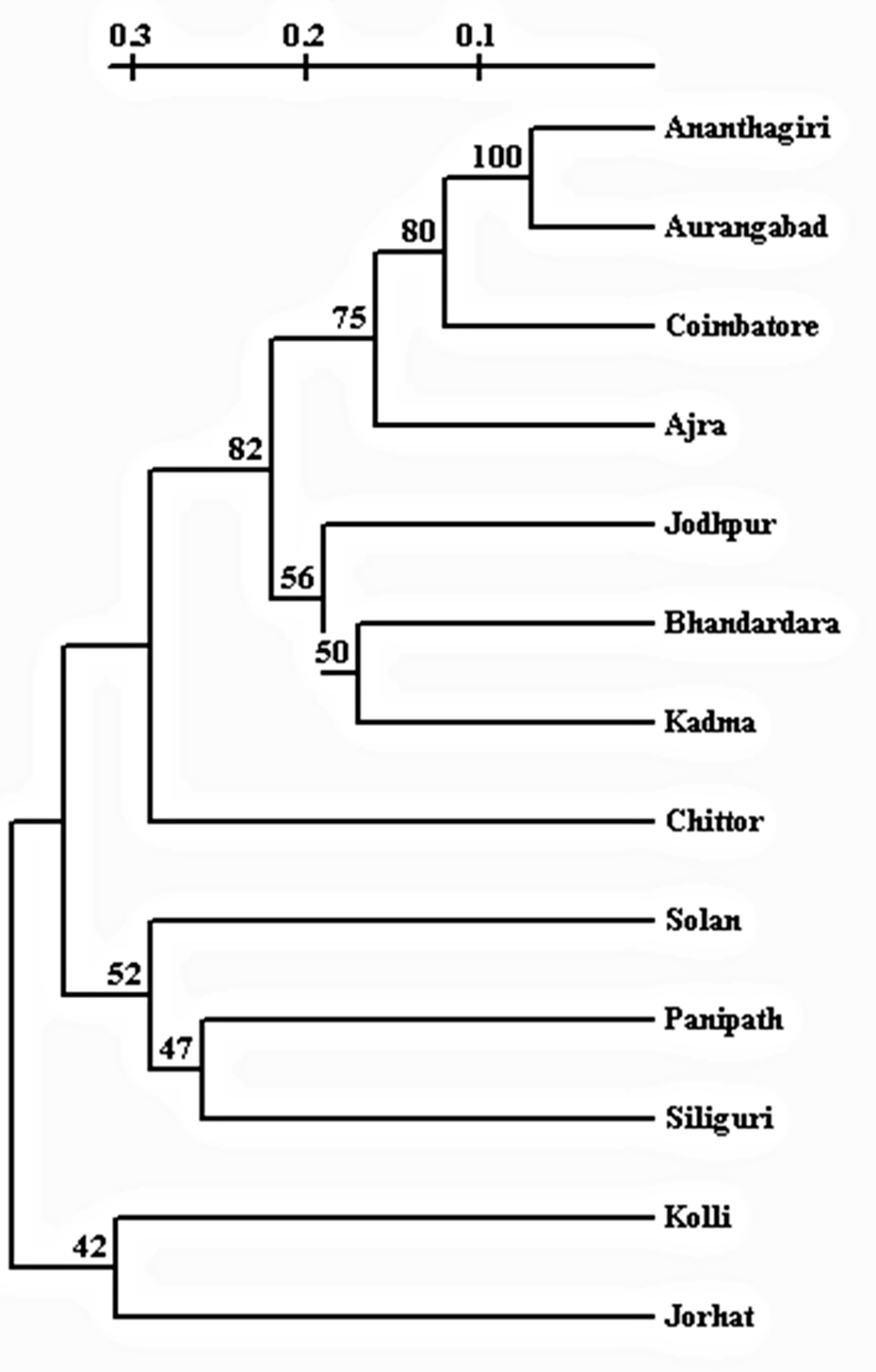


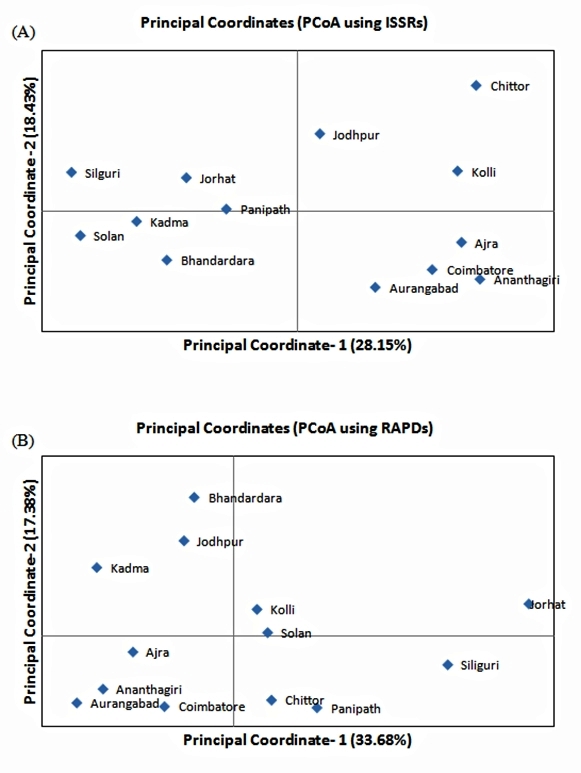


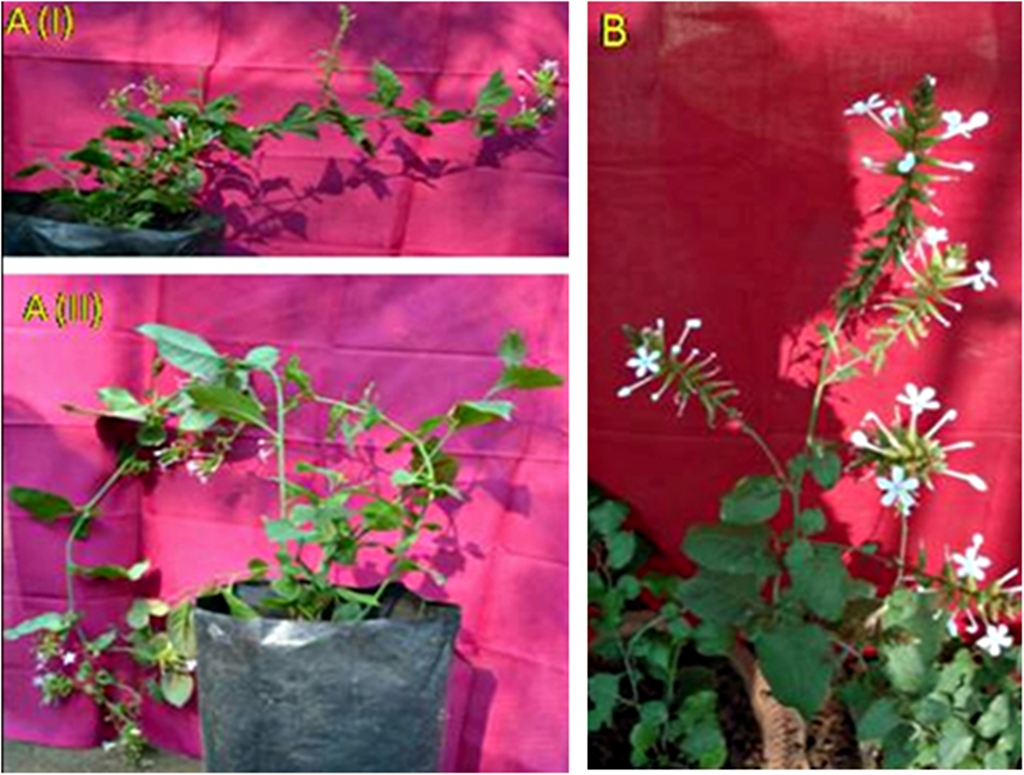

Supplement: Additional Information [file supp_plv048_plv048supp_figs.doc]
