# Supplementary material for: Population structure and genetic diversity of the perennial medicinal shrub Plumbago
Source: AoB Plants. 2015 May 8;7:plv048. doi: 10.1093/aobpla/plv048 (PMC4501514; doi:10.1093/aobpla/plv048)
Supplement: Additional Information [file supp_plv048_plv048supp_tables.doc]

**Table S1 Sampling details of *P. zeylanica*** populations in the present study

| Region | Population Code | Locality | Latitude (N^o^) | Longitude (E^o^) | Altitude (m) | Precipitation (cm) | Sample Size |
| --- | --- | --- | --- | --- | --- | --- | --- |
| North | HP | Solan, Himachal Pradesh | 30^o^51 | 076^o^59.50 | 801 | 160 | 10 |
| North | HR | Panipat, Haryana | 29^o^24 | 076^o^59.58 | 219 | 108 | 10 |
| East | JH | RFRI Campus Jorhat, Assam | 26^o^46 | 094^o^17.37 | 097 | 203 | 10 |
| East | NB | NBU campus Silguri, West Bengal | 26^o^42 | 088^o^21.10 | 136 | 330 | 10 |
| West | RJ | JNVU Campus Jodhpur, Rajasthan | 26^o^15 | 073^o^01.60 | 226 | 030 | 10 |
| East | SB | Kadma, Bankura, West Bengal | 22^o^59 | 087^o^00.59 | 132 | 140 | 10 |
| West | AU | Ellora, Aurangabad, Maharashtra | 19^o^50 | 075^o^18.60 | 556 | 057 | 10 |
| West | BH | Shendi, Bhandardara, Maharashtra | 19^o^33 | 073^o^45.10 | 792 | 394 | 10 |
| South | AN | Ananthagiri, Vikarabad, Andhra Pradesh | 17^o^18 | 077^o^51.80 | 668 | 073 | 10 |
| East | AJ | Ajra-Amboli, Kolhapur, Maharashtra | 16^o^07 | 074^o^13.12 | 674 | 131 | 10 |
| South | CH | Tirupati, Chittor, Andhra Pradesh | 13^o^21 | 079^o^11.43 | 363 | 103 | 10 |
| South | KH | Kolli, Salem, Tamilnadu | 11^o^21 | 078^o^15.68 | 262 | 055 | 10 |
| South | CM | Coimbatore, Tamilnadu | 11^o^03 | 076^o^57.02 | 442 | 063 | 10 |

**Table S2 Descriptive statistics of the 13 traits measured on the 390 individuals of thirteen populations collected from the four different regions.**

| Traits measured | Standard Variation | Mean | Minimum | Maximum | CV |
| --- | --- | --- | --- | --- | --- |
| Length of inflorescence | 2.84 | 11.16 | 6 | 20.2 | 25.45 |
| Length of internodes | 1.37 | 4.81 | 2 | 9.2 | 28.48 |
| Number of inflorescence per vine | 1.37 | 3.66 | 1 | 12 | 37.43 |
| Number of flower per inflorescence | 5.39 | 19.47 | 5 | 38 | 27.68 |
| Distance between two adjacent flower | 0.26 | 0.65 | 0.3 | 2 | 40 |
| Length of sepal | 0.16 | 0.99 | 0.55 | 1.4 | 16.16 |
| Length of petal | 0.19 | 0.81 | 0.5 | 1.3 | 23.46 |
| Length of corolla tube | 0.17 | 2.05 | 1.2 | 2.6 | 8.29 |
| Leaf area | 8.14 | 19.32 | 2.3 | 71.6 | 42.13 |
| Leaf petiole length | 0.33 | 1.46 | 0.5 | 3.1 | 22.6 |
| Colour of trichomes on sepal | 0.51 | 0.38 | 0 | 1 | 132.6 |
| Size of flower | 0.52 | 0.46 | 0 | 1 | 112.67 |
| Plant habit | 0.37 | 0.15 | 0 | 1 | 240.5 |

**Table S3** ISSR primers used for ISSR analysis in the present study

| Primer code | Primer Sequence (5’-3’) | No. of fragments scored | No. of polymorphic bands | Percent (% )  polymorphism |
| --- | --- | --- | --- | --- |
| UBC 807 | (AG)_8_T | 12 | 9 | 75.00 |
| UBC 808 | (AG)_8_C | 10 | 8 | 80.00 |
| UBC 809 | (AG)_8_G | 9 | 4 | 44.44 |
| UBC 811 | (GA)_8_C | 12 | 10 | 83.33 |
| UBC 812 | (GA)_8_A | 10 | 5 | 50.00 |
| UBC 814 | (CT)_8_A | 12 | 10 | 83.33 |
| UBC 822 | (TC)_8_A | 8 | 6 | 75.00 |
| UBC 824 | (TC)_8_G | 7 | 4 | 57.14 |
| UBC 835 | (AG)_8_YC | 14 | 14 | 100.0 |
| UBC 836 | (AG)_8_YA | 10 | 5 | 50.00 |
| UBC 841 | (GA)_8_YC | 17 | 15 | 88.23 |
| UBC 842 | (GA)_8_YG | 14 | 11 | 78.57 |
| UBC 844 | (CT)_8_RC | 9 | 6 | 66.67 |
| UBC 848 | (CA)_8_RG | 11 | 9 | 81.89 |
| UBC 849 | (GT)_8_YA | 17 | 13 | 76.47 |
| UBC 860 | (TG)_8_RA | 14 | 10 | 71.43 |
| UBC 862 | (AGC)_6_ | 14 | 8 | 57.14 |
| UBC 868 | (GAA)_6_ | 12 | 8 | 66.67 |
| UBC 873 | (GACA)_4_ | 10 | 10 | 100.0 |
| UBC 876 | (GATA)_2_(GACA)_2_ | 7 | 4 | 57.14 |
| Total |  | 229 | 169 | 73.81 |

Y = (C, T); B = (C, G, T); D = (A, G, T); H = (A, G, T); V = (A, C, G)

**Table S4** RAPD primers used for RAPD analysis in the present study

| Primer code | Primer sequence (5’-3’) | No. of fragments scored | No. of polymorphic bands | Percent (%) polymorphism |
| --- | --- | --- | --- | --- |
| OPI 01 | ACCTGGACAC | 10 | 8 | 80.00 |
| OPI 02 | GGAGGAGAGG | 9 | 6 | 66.67 |
| OPI 03 | CAGAAGCCCA | 15 | 15 | 100.0 |
| OPI 06 | AAGGCGGCAG | 17 | 13 | 76.47 |
| OPI 07 | CAGCGACAAG | 10 | 6 | 60.00 |
| OPI 09 | TGGAGAGCAG | 16 | 15 | 93.75 |
| OPI 12 | AGAGGGCACA | 8 | 3 | 37.50 |
| OPI 13 | CTGGGGCTGA | 11 | 11 | 100.0 |
| OPI 14 | TGACGGCGGT | 13 | 10 | 76.92 |
| OPI 17 | GGTGGTGATG | 16 | 11 | 68.75 |
| OPI 18 | TGCCCAGCCT | 9 | 8 | 88.89 |
| OPI 20 | AAAGTGCGGG | 14 | 12 | 85.71 |
| OPX 01 | AGCGTCACTC | 10 | 7 | 70.00 |
| OPX 04 | GTCCGTACTG | 12 | 10 | 83.33 |
| OPX 06 | GGTGCTCCGT | 9 | 6 | 66.67 |
| OPX 13 | CTGGGCACGA | 13 | 9 | 69.23 |
| 0PX 15 | CCGCTACCGA | 10 | 8 | 80.00 |
| OPN 13 | ACGCCAGAGG | 9 | 8 | 88.89 |
| OPN 19 | ACGGGAGCAA | 13 | 12 | 92.31 |
| OPN 20 | CAGACAAGCC | 8 | 5 | 62.50 |
| Total |  | 232 | 183 | 78.88 |
